# Efficacy and Safety of She Care Juice, an Ayurvedic Polyherbal Oral Formulation, in Women With Polycystic Ovary Syndrome: A Prospective, Open-Label, Single-Arm Study

**DOI:** 10.7759/cureus.115387

**Published:** 2026-08-29

**Authors:** Shrawan Daga, Priyanka Choudhary, Nivedita Verma, Sanjay Mishra

**Affiliations:** 1 Product Development and Innovation, Sandipani Healthcare Private Limited, Jodhpur, IND; 2 Research and Development, Sandipani Healthcare Private Limited, Jodhpur, IND; 3 Obstetrics and Gynecology, Rumedic Hospital, New Delhi, IND; 4 Clinical Research, Rumedic Hospital, New Delhi, IND

**Keywords:** ayurveda, hirsutism, hyperandrogenism, modified ferriman-gallwey score, nutraceutical, polycystic ovarian disease, polycystic ovary syndrome (pcos), polyherbal formulation, quality of life, she care juice

## Abstract

Background

Polycystic ovarian disease (PCOD) is a common endocrine disorder among women of reproductive age and is characterized by hyperandrogenism, menstrual dysfunction, and metabolic abnormalities. Existing therapeutic approaches may be associated with tolerability concerns and often fail to address the broad spectrum of PCOD manifestations. She Care Juice is a polyherbal Ayurvedic oral formulation containing 15 botanical ingredients. This study evaluated the efficacy and safety of She Care Juice in women with PCOD over a 90-day intervention period.

Methods

This prospective, open-label, single-arm, baseline-controlled clinical study enrolled 30 women aged 18-45 years diagnosed with PCOD, fulfilling the Rotterdam diagnostic criteria for polycystic ovary syndrome (PCOS), at a single clinical site in New Delhi, India. Participants received 30 mL of She Care Juice twice daily for 90 days. The primary efficacy outcomes included changes in hirsutism severity assessed using the modified Ferriman-Gallwey (mFG) score, serum hormonal parameters (luteinizing hormone (LH), follicle-stimulating hormone (FSH), LH/FSH ratio, and total testosterone), and menstrual symptom improvement at Day 90. Secondary outcomes included acne severity measured using the Investigator Global Assessment (IGA) scale, anthropometric measurements, quality of life assessed using the Polycystic Ovary Syndrome Health-Related Quality of Life Questionnaire (PCOSQ), and metabolic parameters. Safety was evaluated through adverse event monitoring and laboratory investigations.

Results

Twenty-six participants completed the study. Mean mFG scores decreased significantly from 11.53 ± 2.87 at baseline to 4.88 ± 1.48 at Day 90 (mean change: -6.62 ± 2.35; p < 0.0001), resulting in scores below the diagnostic threshold for hirsutism. Acne severity improved substantially, with all participants achieving clear or almost clear skin by Day 90 (IGA score: 2.20 ± 0.55 vs. 0.54 ± 0.51; mean change: -1.62 ± 0.70; p < 0.001). At Day 90, 92.3% (24/26) of participants demonstrated clinically meaningful improvement in menstrual-related symptoms, including reduced menstrual symptoms and improved menstrual cycle regularity. Significant reductions were observed in body weight (-3.79 ± 1.60 kg), body mass index (-1.38 ± 0.69 kg/m²), and waist circumference (-4.41 ± 3.67 cm) (all p < 0.001). Quality-of-life outcomes improved markedly, with total PCOSQ scores increasing from 81.50 ± 25.87 at baseline to 173.90 ± 77.60 at Day 90 (p < 0.001). Hormonal parameters exhibited numerical improvements; 53.8% (14/26) of participants achieved a ≥20% reduction in the LH/FSH ratio, although overall hormonal changes did not reach statistical significance. No treatment-emergent adverse events (TEAEs) were reported, and all laboratory safety parameters remained within normal reference ranges throughout the study.

Conclusions

She Care Juice administration for 90 days was associated with significant improvements in hirsutism, acne severity, anthropometric measures, and health-related quality of life in women with PCOD, while demonstrating an excellent safety and tolerability profile. These findings suggest potential therapeutic benefits of this polyherbal formulation and warrant confirmation through larger randomized, double-blind, placebo-controlled trials.

## Introduction

Polycystic ovary syndrome (PCOS) is a heterogeneous endocrine disorder that affects women during their reproductive years and represents one of the leading causes of reproductive and metabolic dysfunction. Global prevalence estimates range from approximately 7% to 17%, although the reported frequency varies according to the diagnostic criteria adopted and the characteristics of the study population [1]. Rather than presenting as a single clinical entity, PCOS encompasses a wide range of reproductive, endocrine, metabolic, and psychological abnormalities with considerable inter-individual variability. The Rotterdam consensus, developed by the European Society of Human Reproduction and Embryology (ESHRE) and the American Society for Reproductive Medicine (ASRM), defines PCOS by the presence of at least two of three diagnostic features after exclusion of alternative disorders: oligo- or anovulation, clinical or biochemical evidence of hyperandrogenism, and polycystic ovarian morphology demonstrated by ultrasonography [2].

PCOS is now understood to involve multiple organ systems rather than the ovaries alone. In routine clinical practice, affected women may present with irregular menstrual cycles, excessive terminal hair growth, acne, increased body weight, insulin resistance, infertility, and psychological distress, either individually or in combination. Beyond these immediate clinical manifestations, the disorder is associated with an increased risk of chronic metabolic and cardiovascular complications, including type 2 diabetes mellitus, dyslipidemia, metabolic syndrome, endometrial pathology, and cardiovascular disease, which may persist throughout adult life [2]. Current evidence suggests that these diverse manifestations arise through interactions among genetic susceptibility, endocrine disturbances, insulin resistance, oxidative stress, inflammatory processes, and environmental influences, resulting in a complex and multifactorial disease process.

Management of PCOS generally requires an individualized and multidisciplinary approach because treatment objectives differ according to age, symptom profile, and reproductive intentions. Lifestyle modification remains the foundation of therapy and is frequently combined with pharmacological interventions such as hormonal contraceptives, insulin-sensitizing medications, ovulation induction agents, or anti-androgen therapy when clinically indicated. Although these therapies improve specific manifestations of the disorder, they do not always address the full spectrum of clinical features, and long-term treatment may be limited by adverse effects, patient adherence, or contraindications, particularly in women planning pregnancy [2]. Consequently, complementary healthcare systems, including Ayurveda, are increasingly being explored as adjunctive therapeutic approaches capable of addressing multiple physiological pathways simultaneously while supporting reproductive and metabolic health [3,4].

Within Ayurvedic medicine, disorders resembling PCOS are interpreted as disturbances in the equilibrium of Kapha and Vata doshas accompanied by impaired Agni and dysfunction of the Artavavaha Srotas. Therapeutic management therefore focuses on restoring physiological homeostasis through normalization of metabolic processes, regulation of endocrine function, reduction of inflammation, improvement of reproductive health, and enhancement of tissue nutrition. Several botanicals traditionally employed in Ayurvedic formulations have demonstrated antioxidant, anti-inflammatory, adaptogenic, insulin-sensitizing, and endocrine-modulating activities in experimental and clinical investigations, providing a scientific rationale for their evaluation as complementary interventions in women with PCOS [5,6].

She Care Juice (Krishna's Herbal & Ayurveda, Jodhpur, India) is a proprietary Ayurvedic polyherbal formulation comprising Daru Haldi (*Berberis aristata*), Rasut (*B. aristata*) extract, Adusa (*Adhatoda vasica*), Bel ka Fal (*Aegle marmelos*), Ashok Chaal (*Saraca asoca*), Dhava (*Woodfordia fruticosa*), Kala Jeera (*Carum carvi*), Shunthi (*Zingiber officinale*), Neel Kamal (*Nymphaea stellata*), Amla (*Phyllanthus emblica*), Harad (*Terminalia chebula*), Baheda (*Terminalia bellirica*), Aam ki Gutli (*Mangifera indica *seed), Nagarmotha (*Cyperus rotundus*), and Chirayata (*Swertia chirata*). The composition, traditional rationale, and pharmacological basis of She Care Juice have been reviewed previously, and therefore only a brief overview is provided here [7]. The phytochemical composition and proposed mechanisms of action of the individual botanical constituents have been reviewed previously [7]. Briefly, the formulation combines botanicals with reported antioxidant, anti-inflammatory, insulin-sensitizing, and endocrine-modulating activities that may be relevant to PCOS. Notably, *B. aristata* contains berberine, a bioactive alkaloid that has demonstrated insulin-sensitizing, anti-hyperglycemic, and androgen-lowering effects in women with PCOS. Similarly, *P. emblica* possesses potent antioxidant activity that may mitigate oxidative stress associated with PCOS, while *S. asoca* has been extensively utilized in traditional medicine for the management of menstrual irregularities and female reproductive disorders [4-6].

Although interest in Ayurvedic therapies for the management of PCOS has grown considerably in recent years, high-quality clinical evidence evaluating standardized polyherbal formulations remains scarce. Most available publications consist of case reports, observational research, or small exploratory studies, limiting the strength and generalizability of the existing evidence. Consequently, there is a need for well-designed prospective clinical investigations to establish the therapeutic efficacy and safety of these formulations under standardized conditions [5,6]. In view of this evidence gap, the present prospective, single-arm, proof-of-concept study was undertaken to investigate the clinical effects and safety profile of She Care Juice administered for 90 days in women with PCOS. The study evaluated its impact on key clinical manifestations, including menstrual health, hirsutism, symptom burden, health-related quality of life, and overall tolerability, with the objective of generating preliminary clinical evidence to inform the design of future randomized controlled trials.

## Materials and methods

Study design and ethical considerations

This prospective, single-center, open-label, single-arm, baseline-controlled clinical study was conducted at Rumedic Hospital, New Delhi, India, between August 2025 and March 2026. Participants were recruited using consecutive sampling from women attending the outpatient gynecology clinic during the study period. The study protocol was reviewed and approved by the Good Society Ethical Research (GSER) Independent Ethics Committee (approval no. GSER/2025/BMR-AP/179; dated July 31, 2025) and was prospectively registered with the Clinical Trials Registry - India (CTRI/2025/08/092842) prior to enrolment of the first participant.

The study was conducted in accordance with the ethical principles outlined in the Declaration of Helsinki (2013), the International Council for Harmonisation Guideline for Good Clinical Practice E6(R3) (ICH E6[R3]), the Indian Council of Medical Research (ICMR) National Ethical Guidelines for Biomedical and Health Research Involving Human Participants (2017), and the New Drugs and Clinical Trials Rules, 2019. Written informed consent was obtained from all participants before the initiation of any study-related procedures. A participant flow diagram is presented in Figure 1 to illustrate participant screening, enrollment, follow-up, and analysis.

**Figure 1:**
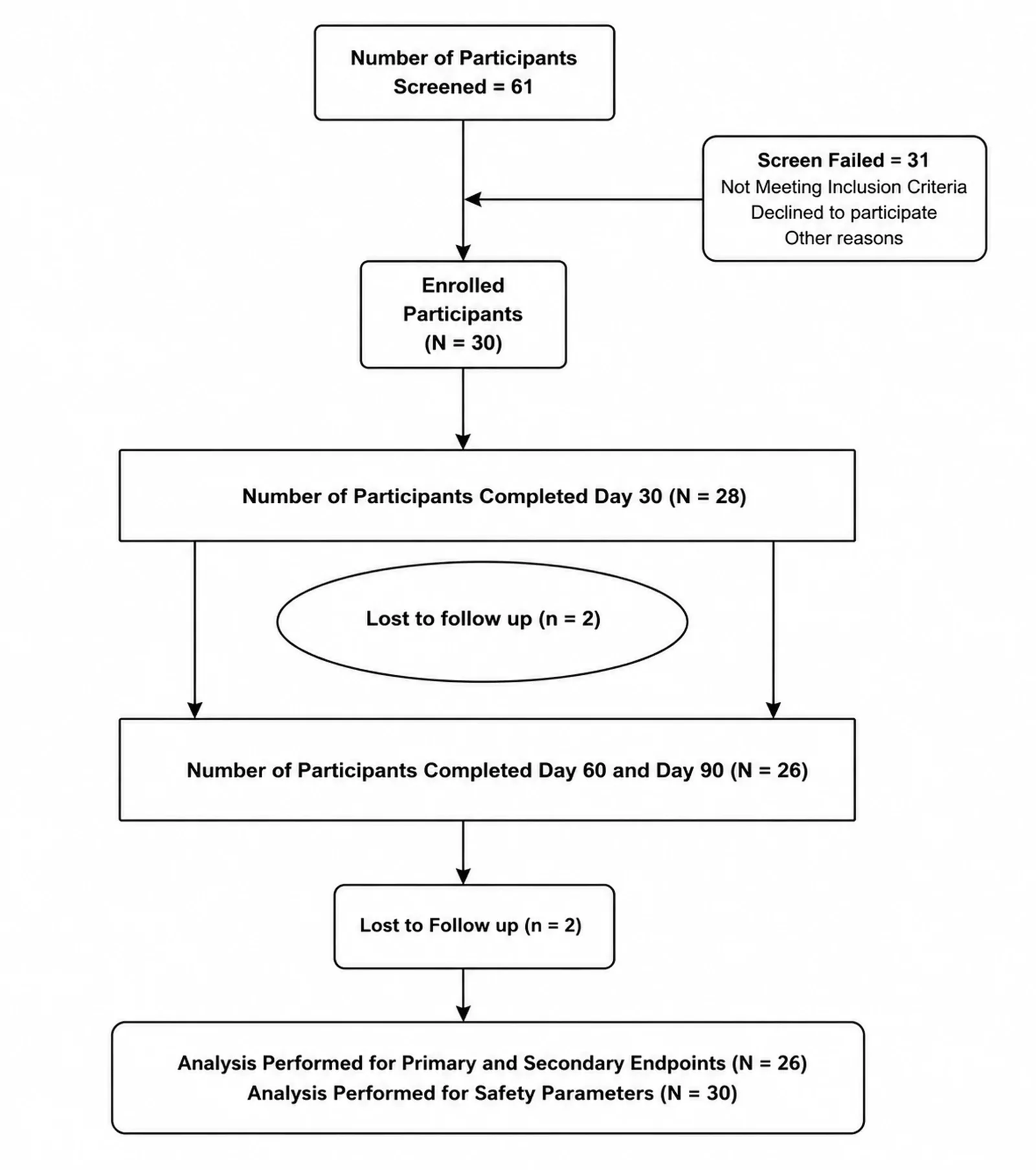
Participant Disposition and Study Flow Flow diagram illustrating participant progression through the study. A total of 61 participants were screened, of whom 30 met the eligibility criteria and were enrolled. Twenty-eight participants completed the Day 30 assessment, while 26 participants completed both the Day 60 and Day 90 visits and were included in the efficacy analyses. Safety analyses were performed on all enrolled participants who received the investigational product (N = 30). Reasons for screen failure included failure to meet inclusion criteria, withdrawal of consent, and other predefined exclusion factors.

Study population

Female participants aged 18-45 years were screened for eligibility. Participants were required to satisfy at least two of the three Rotterdam diagnostic criteria for PCOS, namely, oligo- or anovulation, clinical and/or biochemical hyperandrogenism, and polycystic ovarian morphology on ultrasonography [8]. Additional inclusion criteria included a history of irregular menstrual cycles for at least three to six months, a BMI between 18.5 and 38.0 kg/m², and a luteinizing hormone (LH)/follicle-stimulating hormone (FSH) ratio greater than 1.0.

Participants were excluded if they were pregnant, lactating, or planning pregnancy during the study period; had used hormonal contraceptives or hormone replacement therapy within three months prior to screening; had clinically significant endocrine disorders other than PCOS; had substantial pelvic pathology; or were receiving medications or therapies known to influence hormonal balance. Eligible participants who fulfilled all inclusion and exclusion criteria were enrolled in the study. Potential confounding factors were minimized by prohibiting hormonal therapy, herbal products, nutraceuticals, and medications known to influence endocrine function during the intervention period.

Investigational product and treatment administration

The investigational product, She Care Juice (Krishna's Herbal & Ayurveda, Jodhpur, India), is a polyherbal Ayurvedic formulation manufactured under Good Manufacturing Practice (GMP)-compliant conditions. Prior to release, each batch underwent quality control testing for identity, physicochemical characteristics, microbial contamination, and heavy metal content to ensure product safety and consistency. The formulation contained decoction of *B. aristata* (Daru Haldi), *B. aristata* extract (Rasut), *A. vasica*, *A. marmelos*, *S. asoca*,* W. fruticosa, C. carvi, Z. officinale, N. stellata, P. emblica, T. chebula, T. bellirica, M. indica *seed*, C. rotundus, *and* S. chirata. *Participants received She Care Juice orally at a dose of 30 mL twice daily for 90 consecutive days. They were instructed to consume the first dose in the morning on an empty stomach and the second dose approximately one hour after dinner. The use of concomitant pharmacological agents, nutraceuticals, herbal products, or other interventions known to affect hormonal function was prohibited throughout the study period. Treatment compliance was assessed using participant-maintained daily diary cards and product accountability records. Participants were considered compliant if they consumed at least 80% of the planned study product during the intervention period.

Study procedures and assessments

The study consisted of five scheduled visits, including a Screening visit, Baseline (Day 0), Day 30, Day 60, and the final assessment on Day 90. During the Screening visit, participants were evaluated for eligibility according to the predefined inclusion and exclusion criteria, and written informed consent was obtained prior to the initiation of any study-related procedures. Baseline assessments included demographic data, medical history, physical examination, anthropometric measurements, hormonal and laboratory investigations, and evaluation of clinical symptoms. Follow-up visits on Days 30 and 60 were conducted to assess treatment compliance, monitor clinical progress, record adverse events, and perform scheduled efficacy and safety assessments. The final visit on Day 90 included repeat clinical, laboratory, and patient-reported outcome assessments to determine the efficacy, safety, and overall tolerability of the investigational product following completion of the intervention period.

Primary efficacy assessments

The primary efficacy endpoints included the mean change from baseline to Day 90 in the modified Ferriman-Gallwey (mFG) score, a validated measure of hirsutism severity [8]. Additional primary endpoints included the mean change from baseline in serum LH, FSH, LH/FSH ratio, and total testosterone concentrations at Days 60 and 90 [7]. The proportion of participants reporting clinically meaningful improvement in menstrual-related symptoms at Day 90 was also evaluated.

Secondary efficacy assessments

Secondary efficacy assessments included changes in the Investigator's Global Assessment (IGA) score for acne severity [9], anthropometric parameters, including body weight, BMI, and waist circumference, and health-related quality of life, as assessed using the Polycystic Ovary Syndrome Health-Related Quality of Life Questionnaire (PCOSQ) [10]. Changes in metabolic parameters, including glycated hemoglobin (HbA1c), fasting insulin concentration, Homeostatic Model Assessment of Insulin Resistance (HOMA-IR) index, and lipid profile, were also evaluated during the study period [11,12].

Safety assessments

Safety and tolerability were evaluated throughout the study by continuous monitoring of treatment-emergent adverse events (TEAEs), assessment of vital signs, physical examinations, and serial laboratory investigations conducted at predefined study visits. Participants were questioned regarding the occurrence of any adverse events at each follow-up visit, and all reported events were documented, assessed for severity, duration, outcome, and their potential relationship to the investigational product by the study investigator. Vital signs, including systolic and diastolic blood pressure and pulse rate, were recorded at baseline and at each scheduled follow-up visit. Safety laboratory evaluations included liver function tests (LFTs), renal function tests (RFTs), thyroid function profile, lipid profile, and complete blood count (CBC). Laboratory values were reviewed for clinically significant changes from baseline and compared with normal reference ranges throughout the study period. Any clinically significant abnormalities or unexpected findings were documented, medically evaluated, and managed according to standard clinical practice. Overall tolerability was determined based on adverse event reporting, clinical assessments, treatment compliance, and laboratory safety findings.

Statistical analysis

Statistical analyses were performed using IBM SPSS Statistics for Windows, Version 27.0 (Released 2018; IBM Corp., Armonk, NY, USA). Continuous variables were summarized as mean ± standard deviation (SD), while categorical variables were summarized as frequencies and percentages. Within-group comparisons between baseline and post-treatment assessments were performed using paired t-tests. All statistical tests were two-sided, and a p-value of less than 0.05 was considered statistically significant. Given the exploratory nature of the study, no adjustment for multiple comparisons was performed. No formal sample size calculation was performed because this was an exploratory proof-of-concept study. A target sample size of 30 participants was selected to obtain preliminary estimates of efficacy and safety and to inform future randomized controlled trials. Efficacy analyses were conducted on the per-protocol population, whereas safety analyses included all participants who received at least one dose of the investigational product.

## Results

A total of 61 participants were screened for eligibility, of whom 30 met the inclusion criteria and were enrolled in the study. Twenty-eight participants completed the Day 30 assessment, while 26 participants completed the Day 60 and Day 90 visits and were included in the per-protocol efficacy analysis. Four participants were lost to follow-up during the study period, including two participants after Day 30 and two participants after Day 60. All enrolled participants (N = 30) were included in the safety analysis. Participant disposition throughout the study is presented in Figure 1 and detailed in Table 1. As this was an exploratory proof-of-concept study, a formal power calculation was not performed. A sample size of 30 participants was considered sufficient to evaluate preliminary efficacy signals and safety outcomes.

**Table 1:** Baseline Demographic and Clinical Characteristics (N = 30)

Parameter	Mean (SD)	Range
Age (years)	30.23 (8.08)	18-45
Height (cm)	159.73 (4.88)	148-172
Body weight (kg)	65.42 (10.70)	46-92
Body mass index (BMI) (kg/m²)	25.57 (3.82)	18.5-38.0
Waist circumference (cm)	80.83 (7.78)	65-98
Systolic blood pressure (mmHg)	118.80 (13.44)	77-160
Diastolic blood pressure (mmHg)	75.87 (7.08)	60-90
Pulse rate (beats per minute (bpm))	81.63 (10.21)	64-112
Luteinizing hormone (LH)/follicle-stimulating hormone (FSH) ratio	2.26 (1.70)	1.02-8.10

At baseline, the study population had a mean age of 30.23 ± 8.08 years and a mean height of 159.73 ± 4.88 cm. The mean body weight was 65.42 ± 10.70 kg, with a BMI of 25.57 ± 3.82 kg/m². The mean waist circumference was 80.83 ± 7.78 cm. Baseline hormonal assessment demonstrated a mean LH/FSH ratio of 2.26 ± 1.70. Vital signs were within normal limits at study entry. Baseline demographic and clinical characteristics of the study population are summarized in Table 1.

Effect on hirsutism severity

A statistically significant reduction in hirsutism severity was observed following 90 days of treatment, as assessed using the mFG score. The mean mFG score decreased from 11.53 ± 2.87 at baseline to 4.88 ± 1.48 at Day 90, corresponding to a mean change of -6.62 ± 2.35. The reduction from baseline was highly significant (p < 0.0001). The mean post-treatment mFG score was below the commonly used diagnostic threshold for hirsutism (mFG score ≥8). Detailed numerical results are presented in Table 2. The reduction in mFG score following 90 days of treatment is graphically illustrated in Figure 2.

**Table 2:** Change From Baseline in mFG Score and Serum Hormonal Parameters (N = 26) Paired t-test used for all within-group comparisons. p < 0.05 considered significant.

Parameter	Baseline Mean (SD)	Day 90 Mean (SD)	Change (SD)	% Change	p-value
Modified Ferriman-Gallwey (mFG) score	11.53 (2.87)	4.88 (1.48)	-6.62 (2.35)	-57.4%	< 0.0001
Luteinizing hormone (LH) (IU/L)	15.22 (17.51)	12.38 (12.37)	-3.66 (21.57)	-24.1%	0.396
Follicle-stimulating hormone (FSH) (IU/L)	6.43 (2.88)	6.82 (3.70)	+0.12 (3.83)	+1.9%	0.878
LH/FSH ratio	2.26 (1.70)	1.78 (1.29)	-0.50 (2.05)	-22.1%	0.230
Total testosterone (ng/dL)	25.36 (10.53)	23.83 (12.90)	-1.63 (16.29)	-6.4%	0.613

**Figure 2:**
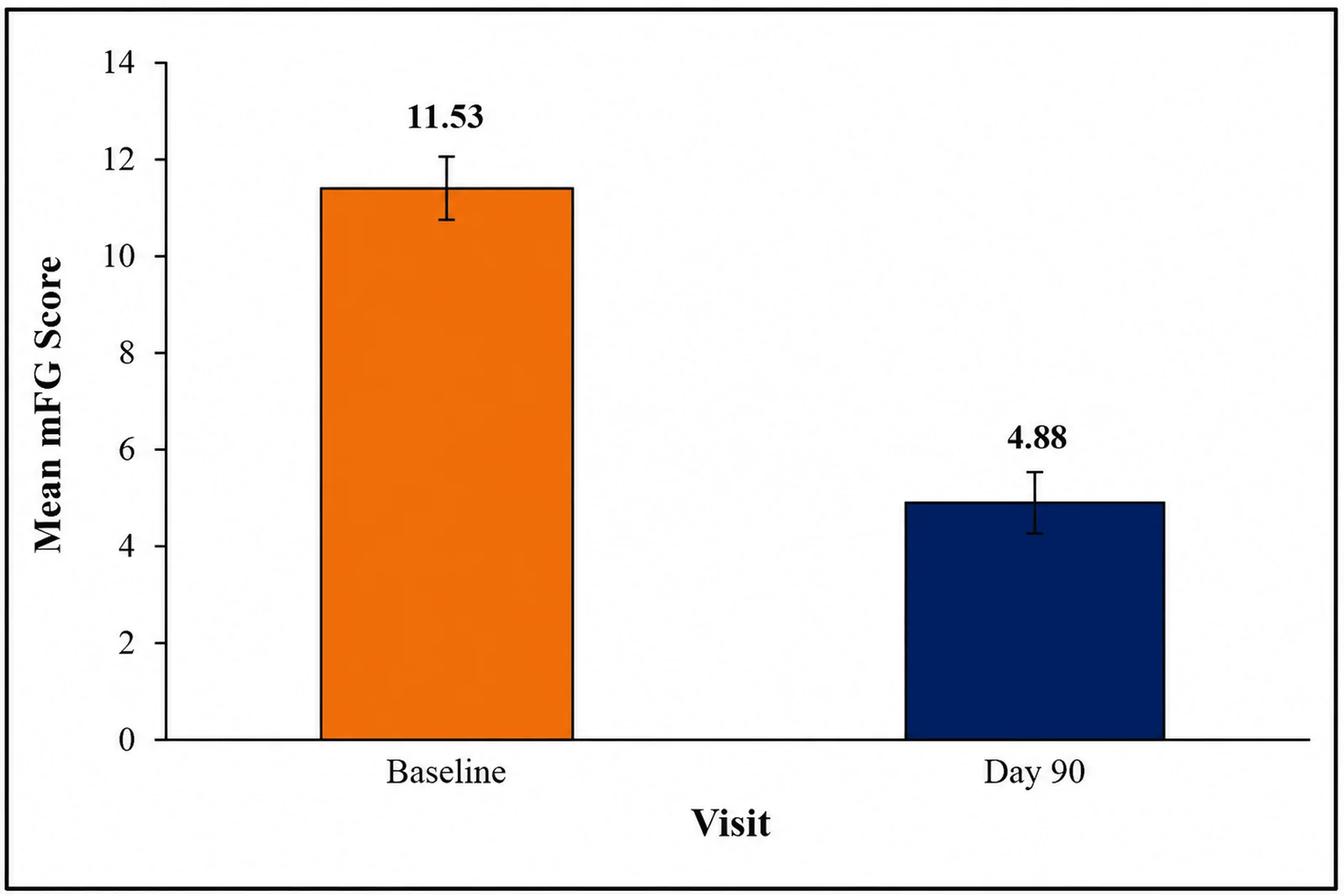
Mean Modified Ferriman-Gallwey (mFG) Score at Baseline and Day 90 Change in mean modified Ferriman-Gallwey (mFG) score from baseline to Day 90. Data are presented as the mean. Percentage change: -57.4% improvement from baseline. Statistical significance between baseline vs Day 90: p < 0.0001. The dashed line indicates the diagnostic threshold for hirsutism (mFG score = 8).

Effect on hormonal parameters

Changes in serum LH, FSH, LH/FSH ratio, and total testosterone concentrations were evaluated at Days 60 and 90. Although directional improvements were observed across these hormonal parameters over the treatment period, the changes from baseline did not achieve statistical significance at the group level. Detailed hormonal outcomes are presented in Table 3.

**Table 3:** Responder Analysis for LH/FSH Ratio Reduction at Day 90 (N = 26) LH/FSH: luteinizing hormone/follicle-stimulating hormone

Response Category	Definition	N	%
Non-responder	No reduction	11	42.3%
Partial response	< 20% reduction	1	3.8%
Responder	20-50% reduction	7	26.9%
Strong responder	> 50% reduction	7	26.9%

Responder analysis of the LH/FSH ratio demonstrated that 53.8% of participants achieved a clinically meaningful reduction of ≥20% from baseline by Day 90. Among these, 26.9% (7/26) of participants were classified as strong responders, exhibiting a reduction of >50% in the LH/FSH ratio. One participant (1/26, 3.8%) demonstrated a partial response (<20% reduction), while 11 participants (11/26, 42.3%) were categorized as non-responders. Improvement in menstrual-related symptoms, assessed using the Menstrual Problems domain of the PCOSQ, was observed in 92.3% (24/26) of participants at Day 90. Of these, 46.2% (12/26) were classified as responders and 46.2% (12/26) as strong responders. No participant was categorized as a non-responder at the end of the study period. Responder analysis for menstrual-related symptom improvement is summarized in Table 4.

**Table 4:** Responder Analysis for Menstrual-Related Symptom Improvement at Day 90 (N = 26)

Response Category	Definition	N	%
Non-responder	Mean domain score <4 (moderately or more bothered)	0	0.0%
Partial response	Mean domain score 4-6 (somewhat to slightly bothered)	2	7.6%
Responder	Mean domain score ≥6 (hardly bothered)	12	46.2%
Strong responder	Mean domain score = 7 (not bothered at all)	12	46.2%

Effect on acne severity

Acne severity, assessed using the IGA score, improved progressively throughout the study period. The mean IGA score decreased from 2.20 ± 0.55 at baseline to 1.50 ± 0.75 at Day 30, 1.27 ± 0.72 at Day 60, and 0.54 ± 0.51 at Day 90. The reductions from baseline at each post-treatment visit were statistically significant (all p < 0.001). By Day 90, 46.2% of participants achieved an IGA score of 0 (clear skin), while the remaining 53.8% (14/26) achieved an IGA score of 1 (almost clear skin). No participant remained in the mild or moderate acne categories at the end of treatment. Detailed changes in IGA scores over time are presented in Table 5.

**Table 5:** Changes in IGA Acne Score and Anthropometric Parameters From Baseline (N = 26) Paired t-test used for within-group comparisons.

Parameter	Baseline Mean (SD)	Day 30 Mean (SD)	Day 60 Mean (SD)	Day 90 Mean (SD)	p-value (Day 90)
Investigator Global Assessment (IGA) score	2.20 (0.55)	1.50 (0.75)	1.27 (0.72)	0.54 (0.51)	< 0.001
Weight (kg)	65.42 (10.70)	64.27 (10.73)	63.66 (10.46)	62.27 (10.02)	< 0.001
BMI (kg/m²)	25.57 (3.82)	25.05 (3.74)	24.78 (3.67)	24.28 (3.51)	< 0.001
Waist circumference (cm)	80.83 (7.78)	79.45 (7.05)	78.58 (6.21)	76.94 (5.68)	< 0.001

Effect on anthropometric parameters

Anthropometric parameters demonstrated progressive improvement throughout the study period. By Day 90, mean body weight had decreased by 3.79 ± 1.60 kg from baseline, while BMI and waist circumference decreased by 1.38 ± 0.69 kg/m² and 4.41 ± 3.67 cm, respectively. Reductions in all three parameters were statistically significant compared with baseline (all p < 0.001). Detailed anthropometric outcomes are presented in Table 5, and comparisons between baseline and Day 90 values are illustrated in Figure 3.

**Figure 3:**
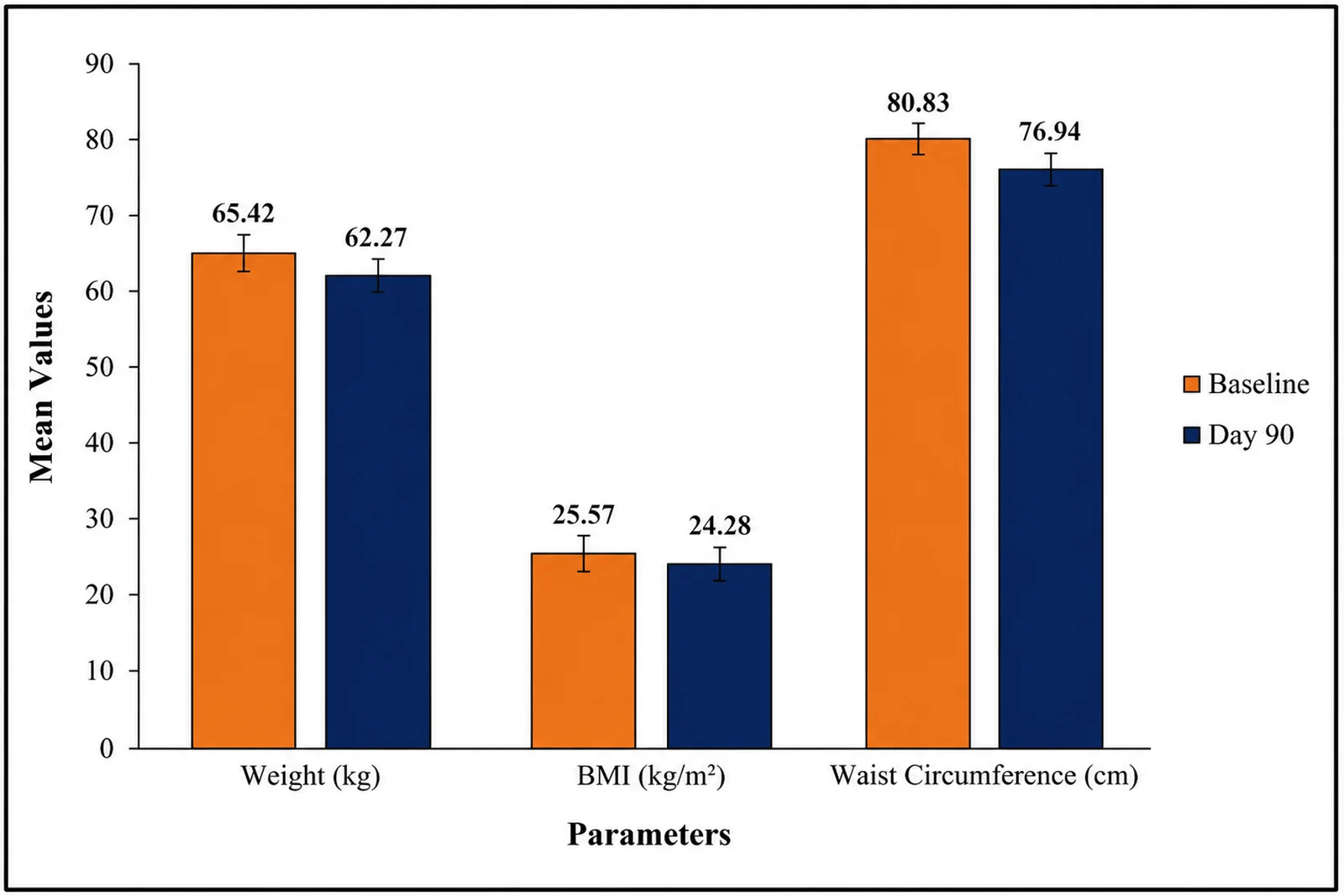
Comparison of Anthropometric Parameters at Baseline and Day 90 Mean body weight, body mass index (BMI), and waist circumference at Baseline and Day 90. Significant reductions were observed in all anthropometric parameters following 90 days of treatment (all p < 0.001).

Effect on health-related quality of life

Health-related quality of life, assessed using the PCOSQ, improved across multiple domains during the study period. The mean total PCOSQ score increased from 81.50 ± 25.87 at baseline to 173.90 ± 77.60 at Day 90 (p < 0.001). Significant improvements were observed in the Emotions, Body Hair, Weight, and Menstrual Problems domains (all p < 0.001). The mean Emotions domain score increased from 19.47 ± 12.12 to 72.43 ± 46.19, the Body Hair domain score increased from 12.60 ± 6.06 to 27.63 ± 11.11, the Weight domain score increased from 12.30 ± 6.68 to 27.33 ± 10.97, and the Menstrual Problems domain score increased from 9.37 ± 5.34 to 22.23 ± 8.96. No change was observed in the Infertility domain score during the study period (p = 1.000). Detailed PCOSQ outcomes are summarized in Table 6.

**Table 6:** Polycystic Ovary Syndrome Health-Related Quality of Life Questionnaire (PCOSQ) Domain and Total Scores From Baseline to Day 90 (N = 26) Higher scores indicate lesser symptom burden. A paired t-test was used.

Domain	Baseline Mean (SD)	Day 90 Mean (SD)	p-value
Emotions	19.47 (12.12)	72.43 (46.19)	< 0.001
Body Hair	12.60 (6.06)	27.63 (11.11)	< 0.001
Infertility	28.00 (0.90)	28.00 (0.90)	1.000
Weight	12.30 (6.68)	27.33 (10.97)	< 0.001
Menstrual Problems	9.37 (5.34)	22.23 (8.96)	< 0.001
Total Score	81.50 (25.87)	173.90 (77.60)	< 0.001

Metabolic parameters

Metabolic parameters, including HbA1c, fasting insulin concentration, and the HOMA-IR, remained stable throughout the 90-day intervention period. No statistically significant or clinically meaningful differences were observed between baseline and Day 90. These findings suggest that the intervention did not adversely affect metabolic homeostasis over the duration of the study.

Safety and tolerability

Safety assessments demonstrated a favorable tolerability profile throughout the 90-day treatment period. No adverse events or serious adverse events were reported among the 30 enrolled participants. Vital signs remained stable, with only minor changes observed during follow-up visits.

Clinical laboratory evaluations, including LFTs, RFTs, thyroid function parameters, hematological indices, and lipid profile, remained within normal physiological reference ranges throughout the study. No clinically significant abnormalities or evidence of treatment-related organ toxicity were observed. Safety laboratory findings are summarized in Table 7.

**Table 7:** Safety Laboratory Parameters at Baseline and Day 90 (N = 30) All parameters remained within normal physiological reference ranges.

Parameter	Baseline Mean (SD)	Day 90 Mean (SD)	Assessment
Liver function tests			
Aspartate Aminotransferase (AST) (U/L)	31.47 (18.37)	34.12 (29.48)	Stable
Alanine Aminotransferase (ALT) (U/L)	27.73 (16.77)	28.29 (23.80)	Stable
Total bilirubin (mg/dL)	0.570 (0.269)	0.535 (0.320)	Stable
Renal function tests			
Serum creatinine (mg/dL)	0.571 (0.106)	0.544 (0.096)	Stable
Blood urea (mg/dL)	20.57 (7.04)	22.62 (4.37)	Stable
Thyroid function			
Thyroid-Stimulating Hormone (TSH) (µIU/mL)	3.407 (2.05)	2.988 (2.09)	Stable
Hematology			
Hemoglobin (g/dL)	11.72 (1.57)	11.80 (1.95)	Stable
Platelet count (×10⁵/µL)	2.274 (0.730)	2.497 (0.758)	Stable

## Discussion

The present study provides preliminary clinical evidence that supplementation with She Care Juice may improve several clinically relevant manifestations of PCOS over a 90-day treatment period. Improvements were observed across hyperandrogenic symptoms, anthropometric measures, menstrual health, and patient-reported quality of life, while the formulation was well tolerated throughout the intervention. The pharmacological rationale of the formulation has been discussed in detail previously [7]; therefore, the present discussion focuses primarily on interpretation of the clinical findings. Although the uncontrolled study design precludes definitive conclusions regarding treatment efficacy, the consistent improvements observed across multiple clinical outcomes support further investigation of this standardized Ayurvedic formulation in adequately powered randomized clinical trials [13,14].

A notable finding of this study was the marked reduction in hirsutism severity after three months of supplementation. The average mFG score declined from values consistent with clinical hirsutism to levels below the commonly accepted diagnostic threshold, indicating improvement in one of the most distressing manifestations of PCOS. Because excessive terminal hair growth develops gradually and typically responds slowly to treatment, the extent of improvement observed within a relatively short intervention period is encouraging. Since hirsutism is closely associated with hyperandrogenism and psychological well-being, improvement in this outcome may contribute to enhanced patient confidence and overall quality of life [2,15].

Progressive improvement in acne severity was observed throughout the intervention, with all participants achieving either clear or almost clear skin at the final assessment. Because acne and hirsutism arise from overlapping endocrine disturbances, particularly increased androgen activity and chronic low-grade inflammation, concurrent improvement in both outcomes may indicate that the intervention influences multiple biological pathways involved in PCOS [10,16]. Previous experimental and clinical investigations have reported antioxidant, anti-inflammatory, endocrine-modulating, and metabolic regulatory activities for several botanical constituents of She Care Juice [7]. These observations provide biological plausibility for the clinical responses observed in the present study but do not establish causality.

Significant reductions in body weight, BMI, and waist circumference further strengthen the clinical relevance of the present findings. Excess adiposity is recognized as an important contributor to insulin resistance, endocrine dysfunction, and chronic low-grade inflammation in women with PCOS. Previous clinical guidelines have demonstrated that even moderate reductions in body weight can improve metabolic and reproductive outcomes in this population [16]. Therefore, the anthropometric improvements observed during this study may reflect broader metabolic benefits extending beyond simple weight reduction. From an Ayurvedic perspective, these changes may correspond to improved metabolic regulation through restoration of digestive function and normalization of physiological balance [3,4].

Although favorable numerical changes were observed in endocrine biomarkers following supplementation, statistically significant differences were not detected for LH, FSH, the LH/FSH ratio, or total testosterone. This pattern is not unexpected in exploratory clinical studies because hormonal responses in women with PCOS are highly heterogeneous and may require longer treatment duration before measurable biochemical changes become apparent [5,14]. Notably, responder analysis suggested that a considerable proportion of participants experienced clinically relevant reductions in the LH/FSH ratio, indicating heterogeneity in treatment response. Consequently, the absence of statistically significant changes at the group level should not be interpreted as evidence of a lack of biological activity but rather as a reflection of biological variability, limited statistical power, and the multifactorial pathophysiology of PCOS, which extends beyond isolated endocrine markers [16].

Improvement in menstrual-related symptoms represented another important clinical outcome. More than 90% of participants reported clinically meaningful improvement in menstrual health following treatment. Although ovulation and menstrual cyclicity were not objectively assessed, patient-reported improvements suggest a potential beneficial effect on reproductive function. Restoration of menstrual regularity is a major therapeutic objective in women with PCOS because it reflects improved endocrine regulation and may ultimately contribute to better reproductive health. These preliminary observations should be confirmed using objective reproductive endpoints in future controlled studies [16].

Improvements in quality of life closely paralleled the observed clinical benefits. Significant increases in PCOSQ scores were evident across the emotional, body hair, body weight, and menstrual domains, indicating that symptom improvement translated into meaningful benefits from the patient's perspective. Since PCOS frequently affects psychological well-being in addition to physical health, incorporating patient-reported outcomes provides a more comprehensive evaluation of therapeutic benefit. The absence of change within the infertility domain was expected because fertility-related outcomes were beyond the objectives of the present study and baseline scores in this domain were already high [11].

No safety signals attributable to the investigational product were identified during the intervention period. Clinical laboratory parameters, including hepatic, renal, thyroid, hematological, and lipid assessments, remained within normal reference limits, and no participant experienced a TEAE. These findings indicate that the formulation was well tolerated during the three-month supplementation period. Given that long-term pharmacological therapy for PCOS may be associated with adverse effects or reduced adherence in some patients, complementary interventions demonstrating favorable tolerability may represent valuable additions to existing management strategies [2].

Several limitations should be considered when interpreting these findings. The absence of a placebo or active comparator limits attribution of the observed improvements exclusively to the investigational product. In addition, the relatively small sample size, single-center setting, and short duration of follow-up restrict the generalizability of the results. Objective assessment of ovulatory function, menstrual cyclicity, fertility outcomes, and longer-term metabolic changes was not incorporated into the study protocol. Consequently, the present findings should be regarded as preliminary evidence requiring confirmation in larger, well-controlled clinical investigations.

Despite these limitations, the study contributes important preliminary clinical evidence regarding the potential role of a standardized Ayurvedic polyherbal formulation in the management of PCOS. Taken together, the findings suggest that the formulation may influence multiple clinical domains of PCOS simultaneously rather than targeting a single manifestation of the disorder. This multi-dimensional pattern of response supports further investigation of standardized Ayurvedic formulations within evidence-based integrative management strategies. Future multicenter randomized trials incorporating mechanistic biomarkers, reproductive outcomes, and longer follow-up periods will be important for establishing clinical effectiveness and identifying the biological pathways responsible for the observed improvements.

## Conclusions

Daily supplementation with She Care Juice for 90 days was associated with clinically relevant improvements in multiple manifestations of PCOS, including hirsutism, acne severity, anthropometric parameters, menstrual-related symptoms, and patient-reported quality of life. The formulation demonstrated a favorable safety profile, with no TEAEs or clinically significant laboratory abnormalities observed during the intervention period. Although hormonal biomarkers showed only modest numerical changes, the overall pattern of clinical improvement suggests that this standardized Ayurvedic polyherbal formulation may provide adjunctive therapeutic benefits as part of an integrative approach to PCOS management. Future adequately powered randomized controlled trials should determine whether these encouraging preliminary observations translate into sustained clinical and reproductive benefits across diverse patient populations.
